# Percutaneous endoscopic lumbar discectomy: clinical and quality of life outcomes with a minimum 2 year follow-up

**DOI:** 10.1186/1749-799X-4-20

**Published:** 2009-06-25

**Authors:** Chan WB Peng, William Yeo, Seang B Tan

**Affiliations:** 1 Department of Orthopedic Surgery, Singapore General Hospital, Outram Road, Singapore 169608, Singapore; 2 Department of Physiotherapy, Singapore General Hospital, Outram Road, Singapore 169608, Singapore

## Abstract

**Background:**

Percutaneous endoscopic lumbar discectomy is a relatively new technique. Very few studies have reported the clinical outcome of percutaneous endoscopic discectomy in terms of quality of life and return to work.

**Method:**

55 patients with percutaneous endoscopic lumbar discectomy done from 2002 to 2006 had their clinical outcomes reviewed in terms of the North American Spine Score (NASS), Medical Outcomes Study Short Form-36 scores (SF-36) and Pain Visual Analogue Scale (VAS) and return to work.

**Results:**

The mean age was 35.6 years, the mean operative time was 55.8 minutes and the mean length of follow-up was 3.4 years. The mean hospital stay for endoscopic discectomy was 17.3 hours. There was significant reduction in the severity of back pain and lower limb symptoms (NASS and VAS, p < 0.05) at 6 months and 2 years. There was significant improvement in all aspects of the Quality of Life (SF-36, p < 0.05) scores except for general health at 6 months and 2 years postoperation. The recurrence rate was 5% (3 patients). 5% (3 patients) subsequently underwent lumbar fusion for persistent back pain. All patients returned to their previous occupation after surgery at a mean time of 24.3 days.

**Conclusion:**

Percutaneous endoscopic lumbar discectomy is associated with improvement in back pain and lower limb symptoms postoperation which translates to improvement in quality of life. It has the advantage that it can be performed on a day case basis with short length of hospitalization and early return to work thus improving quality of life earlier.

## Introduction

The surgical treatment of lumbar disc herniation constitutes a large part of orthopedic practice and it has evolved considerably in terms of surgical technique and instrumentation.

Percutaneous endoscopic discectomy is a relatively new technique for removing lumbar disc herniation. It involves using an endoscope to visualize the disc removal. The discectomy is performed through a posterolateral approach using specially developed instruments. The advantage of percutaneous endoscopic discectomy is that the disc is approached posterolaterally through the triangle of Kambin [1,2] without the need for bone or facet resection thus preserving spinal stability. [1-4] There is less damage to muscular and ligamentous structures allowing for faster rehabilitation, shorter hospital stay and earlier return to function.

Although many studies [1-8] have shown the efficacy of percutaneous endoscopic discectomy with good clinical outcomes, there are very limited reports of how this translates to quality of life improvement and ability to return to work. Health-related quality of life measures that are patient-oriented (self-administered questionnaire) are important in evaluating neurologic and spinal disorders, especially since they affect the general status of the patients. It has even been suggested that more widespread use of standardized health measures may improve clinical practice. [9,10]

The purpose of this study is to determine the outcome of percutaneous endoscopic discectomy in terms of the North American Spine Score (NASS) [11], Medical Outcomes Study Short Form-36 'Quality of Health' scores (SF-36) [9,10] and Pain Visual Analogue Scale (VAS) and how well patients have returned to work.

## Methods

From 2002 to 2006, 55 patients with percutaneous endoscopic discectomy performed for herniated intervertebral disc at our instituition had data collected prospectively. All the operations were performed by two surgeons.

Inclusion criteria were patients who had radicular symptoms due to discogenic lumbar nerve root compression and failed conservative therapy. The diagnosis of lumbar disc herniation was made on MRI and/or CT scans. Patients with calcified discs shown on CT scans were excluded. Patients who met the inclusion criteria were counseled that percutaneous endoscopic lumbar discectomy was a relatively new technique and offered the alternative of open discectomy as well. 55 patients agreed to have percutaneous endoscopic discectomy.

Data on patient demographics, operative time, length of hospitalisation, postoperative complications and how soon they returned to work were obtained. In our institution, all patients who underwent spinal surgery had routine preoperative assessment and 6 month and 2 year postoperative assessments done; the patients were assessed based on the North American Spine Score (NASS – Disease specific questionnaire) [11], Medical Outcomes Study Short Form-36 scores (SF-36 – Quality of life questionnaire) [9,10] and Visual Analogue Scale (VAS) for pain (Table 1). All patients were evaluated by an independent observer not involved in the surgical procedure.

**Table 1:** Patient demographics, operative time, length of hospital stay and duration of followup for endoscopic discectomy

	**Endoscopic discectomy**
Age	Mean 35.6 years
	Range 15 – 68 years
Sex	23 female: 32 male
	42% female: 58% male
Operative time	Mean 55.8 minutes
	Range 30 – 100 minutes
Length of hospital stay	Mean 17.3 hours
	Range 6 to 24 hours
Follow-up	Mean 3.4 years
	Range 2.0 – 6.5 years
Duration of medical leave	Mean 24.3 days
	Range 10 – 60 days

Statistical analysis was performed with the use of SPSS version 10.0. Categorical data were compared with the use of chi-square test. Non-parametric statistics were used for the analysis of continuous variables when data were not normally distributed. Significance was defined as p < 0.05.

### Technique

Preoperatively, all patients received one gram of cefazolin intravenously as antibiotic prophylaxis and if the patient is allergic to cefazolin, one gram of intravenous vancomycin was given instead. The patients were placed prone on a radiolucent operative table on a Wilson frame.

36 of the 55 patients (66%) were done under local anesthesia. The skin, subcutaneous tissue, fascia and muscle layers were infiltrated with 1 per cent lidocaine. For relaxation and comfort of the patient, sedation with intravenous midazolam or Fentanyl was administered by the anesthetist. 19 patients (34%) were uneasy about having the operation performed under local anaesthesia and so the operation was done under general anaesthesia.

Using the C-arm oriented in the postero-anterior imaging position, the midline longitudinal line is marked on the skin surface using a narrow metal rod. The metal rod is then placed transversely across the center of the target disc. A horizontal line is drawn, bisecting the disc under evaluation. The anatomic disc center is located where the transverse line crosses the longitudinal midline. The C-arm is rotated to the lateral projection. The metal rod is held along the side of the patient in the parasagittal orientation at the level of the index disc. While the metal rod is held in this position, the length from the center of that disc to the plane of the posterior skin is recorded. This length is used for the lateral distance of the skin entry point from the posterior midline and this is usually about 12 to 14 cm from the midline. [12] Under fluoroscopic guidance, an 18 gauge spinal needle is inserted such that the needle tip is positioned at the medial pedicular line in the anteroposterior projection and on the posterior vertebral line in the lateral projection. In patients done under local anaesthetic, a transforaminal epidural infiltration with 1% lidocaine is injected through the spinal needle to reduce pain and discomfort. The needle is then punctured into the disc and an intraoperative discogram is performed with a mixture of 6 ml of contrast media and 1 ml of indigo carmine. The indigo carmine stains the pathologic nucleus and the annular fissure for easy discrimination through the endoscope.

A guidewire is then inserted through the needle into the disc and the needle removed. A small stab incision is made at the entry site of the guidewire and a tapered cannulated obturator is slid over the guide wire and introduced gently into the foramen and into the disc. A beveled working cannula is then introduced over the obturator which is then withdrawn. An endoscope (Yeung Endoscopic Spine System – Y.E.S.S. endoscope) [13] is then inserted through the working channel and discectomy performed with endoscopic forceps. Discectomy is performed to a location just under the apex of the herniation. An endoscopic rongeur is used to extract the blue-stained material creating a cavity within the disc. If a noncontained extruded disc fragment is present, the anular collar is divided, and a cutting forceps is used to perform a partial anulectomy. Once a partial anulectomy has been carried out, the subligamentous or extraligamentous components of the herniation are first extracted into the cavity within the disc and then pulled out through the endoscope working channel. Hemostasis is performed with bipolar diathermy (Ellman International, Hewlett, NY) [13].

## Results

The mean age of the patients was 35.6 years (range 15 – 68 years). There were 23 (41.8%) female: 32 (58.2%) male. The mean operative time was 55.8 min (30–100 min). The mean length of hospitalization was 17.3 hours (range 6 to 24 hours). The mean follow-up period was 3.4 years (range 2.0 – 6.5 years). All patients who were working preoperatively returned to work. The mean time to return to work was 24.3 days (10 – 60 days). All returned to their previous occupation (Table 1).

39 (70.9%) patients had L4L5 discectomy done, 12 (21.8%) had L5S1, 2 (3.6%) had L3L4 and 2 (3.6%) had two levels L4L5 and L5S1 done. There were 44 (80%) disc protrusions, 10 (18.2%) extrusions and 1 (1.8%) sequestrated disc.

**Figure 1:**
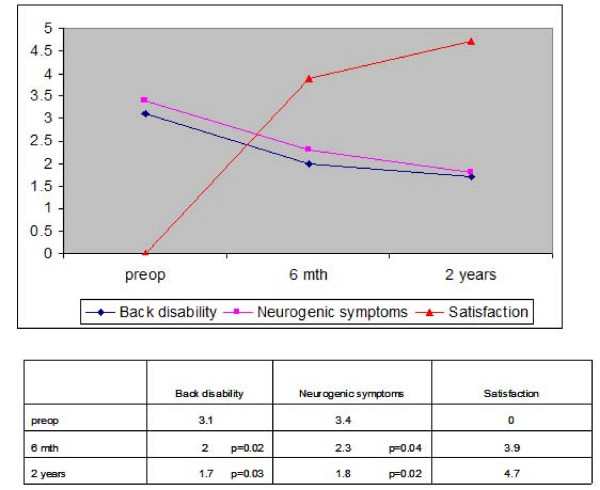
**NASS scores pre and postoperatively**.

**Figure 2:**
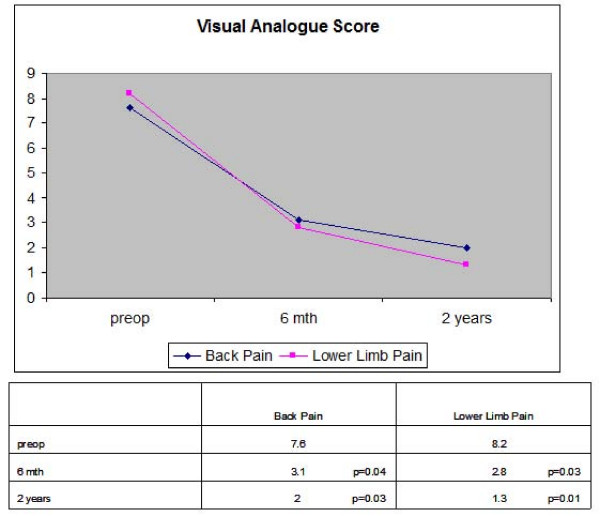
**Visual Analogue Scale pre and postoperatively**.

Figure 1 and 2 shows the preoperative and 6 month and 2 years postoperative NASS and VAS scores. There was significant improvement in the NASS scores for back disability and neurogenic symptoms and the VAS scores for back pain and lower limb pain at 6 months and 2 years postoperatively compared to preoperatively (all p < 0.05). The mean NASS score for satisfaction with treatment was 3.9 (range 1.3–5.4) at 6 months and 4.7 (range 2.5 – 5.8) at 2 years postoperation (1 = extremely dissatisfied, 6 = extremely satisfied). Low satisfaction scores were reported by patients who had complications or required subsequent operations. These included 3 patients who developed recurrent disc prolapse, 3 patients who underwent subsequent lumbar fusion for increasing back pain and 1 patient who developed post-operative discitis. 3 patients had recurrent disc prolapse (recurrence rate 5%). All these patients had relief of their leg symptoms after the operation. One patient had recurrence of symptoms at 6 weeks post-operation, another at 4 months and the third at 7 months post-operation. 2 of these patients subsequently underwent open discectomy. Both had relief of symptoms with no complications after the open discectomy. One patient with recurrent disc refused operation and was treated conservatively. 1 patient had a sequestrated disc after endoscopic discectomy and was treated with open discectomy.

3 patients (5%) subsequently underwent lumbar fusion for increasing back pain despite good relief of radicular symptoms after endoscopic discectomy. One of these patients was a 25 year old male who presented initially with left L4 radicular symptoms. MRI showed L4L5 and L5S1 degenerate discs with a left L4L5 prolapsed intervetebral disc. A left L4L5 endoscopic discectomy was initially performed for him but on followup, he complained of increasing back pain and had L4L5, L5S1 transforaminal lumbar interbody fusion done 3 months after endoscopic discectomy. Another was a 45 year old male who had right L5 radicular symptoms and back pain preoperatively. MRI showed a right L5S1 posterolateral disc prolapse. He underwent right L5S1 endoscopic discectomy but also had increasing back pain on followup. He eventually had L5S1 posterior lumbar interbody fusion done 7 months post-edoscopic discectomy. For both of these patients, their initial radicular symptoms resolved after endoscopic discectomy. The third patient was a 36 year old female with left L4 radicular pain. MRI showed a L4L5 prolapsed disc. Post- endoscopic discectomy, her radicular sumptoms resolved. However 3 years postoperation, she complained of back pain and left L4 radicular pain again. Postoperation MRI showed diffuse L45 disc bulge and central and lateral recess stenosis. She subsequently underwent transforaminal lumbar interbody fusion.

1 patient developed discitis 4 days post-endoscopic discectomy. This is a 37 year old male who underwent left L45 percutaneous endoscopic discectomy. He was discharged well 1 day post-operation. However, on the 4^th ^post-operative day, he complained of severe back pain associated with mild fever. Blood tests showed raised total white count, ESR and CRP. An MRI with contrast showed mainly granulation tissue. He underwent endoscopic washout of the disc space. Tissue cultures from the disc space grew *Staphylococcus aureus*. His symptoms resolved after the washout. He was treated with intravenous Augmentin for 2 weeks followed by a further 4 weeks of oral Augmentin. He had occasional back pain at 2 years follow-up. There were no complications associated with any of the subsequent surgeries performed after endoscopic discectomy.

**Figure 3:**
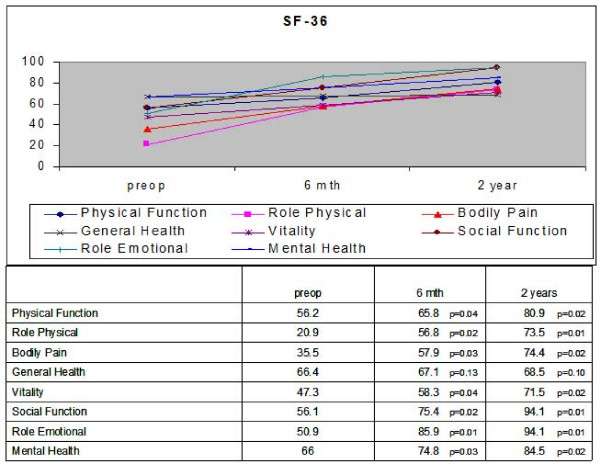
**SF-36 scores pre and postoperatively**.

Based on the SF-36 questionnaire (Figure 3), all aspects of 'Quality of Life' improved after endoscopic discectomy. At 6 months and 2 years post operation, there was significant improvement in scores for physical function, role physical, bodily pain, vitality, social function, role emotional and mental health (all p < 0.05). However the improvement in general health scores did not reach significant difference at 6 months and 2 years postoperation.

## Discussion

Conventional open surgery remains the 'gold standard' for treating herniated intervertebral disc. However the disadvantages of open surgery include extensive retraction and dissection of paraspinal muscles, longer operative time, larger wounds and bone resection. [5,14]

Endoscopic discectomy via a percutaneous transforaminal posterolateral approach is an alternative technique used to treat lumbar disc herniations. Advances in instrumentation now allow for a 'working channel' through which various tools can be passed under direct endoscopic visualization for the safe removal of disc material. The advantages of this technique include less paraspinal musculature trauma and smaller wounds. Bone removal is not required to decompress the exiting nerve root and this avoids the risk of inducing spinal instability. [2,3,15] Also the spinal canal is not violated and therefore there is less epidural bleeding and epidural scarring. However, unlike other percutaneous techniques like chymopapain chemonucleolysis, percutaneous laser discectomy and nucleoplasty, percutaneous endoscopic discectomy allows removal of not only fragments located in the center of the nucleus, but also fragments that have migrated posteriorly and posteroaterally by using specially designed straight, upbiting and deflectable forceps under endoscopic control.

Many studies have shown good to excellent clinical outcomes after percutaneous endoscopic discectomy based on improvement in disease-related symptoms and physical signs. [3-8] However, these are surgeon-based outcome measures which are not related with validated measurements of outcomes that are more relevant to patients' quality of life and functional status. These measures place no emphasis on the patient's overall perception of the impact of the operation on subjectively experienced distress or well-being. To assess the impact on quality of life in patients who undergo percutaneous endoscopic discectomy, the SF-36 questionnaire was administered to our patients preoperatively and at 6 months and 2 years postoperation. Based on the SF-36 questionnaire, all aspects of 'Quality of Life' scores improved after endoscopic discectomy compared to preoperation. There was also significant improvement in NASS and VAS scores at 6 months and 2 years postoperation compared to preoperation. Thus back pain and neurogenic symptoms are particularly disabling and are associated with significant morbidity affecting quality of life. Hence surgical treatment to improve these symptoms translates to significant improvement in the quality of life of patients.

In our study, the mean hospital stay for endoscopic discectomy was 17.3 hours. Other studies have also shown that endoscopic discectomy can be performed on an outpatient basis and discharged within 24 hours. [14,15] The median hospital stay for patients treated with conventional open discectomy range from 3 to 4 days. [16] Therefore, endoscopic discectomy has the advantage of shorter hospitalization compared to open approaches. In our study the mean time to return to work is 24.3 days and all patients returned to their previous occupation. Other studies also showed that the majority of patients were able to return to their previous occupation within 1 month, and that the period of disability is shorter for endoscopic discectomy compared to open discectomy. [3,5,14] Thus endoscopic discectomy is associated with short hospitalization and earlier return to work and patients can achieve improved quality of life earlier.

Most studies report that performing this procedure under local anesthesia with constant intraoperative feedback from patients is important in reducing the risk of neural damage. [2,3,5-8,15,17] In our study, 66% of our patients were done under local anesthesia and 19 patients were done under general anesthesia. We feel that although the potential risk of nerve damage should be recognized, percutaneous endoscopic discectomy can still be performed safely under general anesthesia as long as the approach to the disc is kept within the triangle of Kambin [2]. However, this requires careful reading of the preoperative imaging studies and intraoperative fluoroscopy. Also when the endoscope is inserted, it is important to examine that the nerve is not entrapped. We felt that if the endoscope is introduced at the safe triangle of Kambin, [2] the risk of nerve damage was low. We did not have any neurological deficit in all the patients done under general anesthesia. The advantage of general anesthesia is that there is no patient discomfort and intraoperative pain that is associated with performing the procedure under local anesthesia.

3 patients had recurrent disc prolapse (recurrence rate 5%), of which 2 had open discectomy. 1 patient was found to have a sequestrated disc post-endoscopic discectomy and had open microdiscectomy subsequently. Recurrence rate of lumbar disc herniation after open discectomy has been reported as 5–11% and most have been treated with a repeated discectomy through the same approach as the initial surgery.[18,19] However, repeat open discectomy through the same initial approach could produce less satisfactory results with approach related complications. Scar tissue from the previous open surgery makes repeat discectomy more difficult with increased risk of dural tear or nerve injury. [20,21] In our study, when open discectomy was performed for recurrent disc prolapse/sequestrated disc after previous endoscopic discectomy, there was no scar tissue encountered and there were no associated complications. In these patients, the endoscopic procedure did not seem to have a disadvantageous influence on the outcome of subsequent open surgery since all had resolution of their symptoms. Other studies [5,15] also showed that successful outcomes can be achieved in repeat operations for failed percutaneous endoscopic discectomy.

3 patients subsequently underwent lumbar intervertebral body fusion for increasing low back pain despite resolution of their initial radicular symptoms. Thus while percutaneous endoscopic discectomy is effective in relieving leg symptoms, it is less effective in treating back pain and this has to be communicated to the patients.

In this study, the complications for endoscopic discectomy include 1 case of discitis and 1 case of sequestrated disc, giving a complication rate of 3.6%. It has been reported that the overall complication rate for this kind of surgical procedure averages 2.6%.[8,22] The complications reported include dysthesia, nerve root or vascular injury, postoperative infections and dural tear.[6,8,13,17,22,23] The incidence of failures and complications in this group of patients was similar to that experienced with conventional open surgery.[24]

## Conclusion

Percutaneous endoscopic lumbar discectomy is a safe and efficacious technique to relieve symptoms of herniated discs and this improvement in back pain and leg symptoms translates to improvement in quality of life. It has the advantage that it can be performed on a day case basis with shorter length of hospitalization and early return to work thus improving quality of life earlier. This is important because patients become candidates for lumbar disc herniation surgery to obtain immediate pain relief and to improve their quality of life.

## Competing interests

The authors declare that they have no competing interests.

## Authors' contributions

CWBP performed case collection, data analysis, literature review and wrote article. WY performed case and data collection. SBT supervised and helped in manuscript preparation. All authors read and approved the final manuscript.
